# Case of Malignant Disease of the Right Upper Jaw. Excision—Recovery

**Published:** 1857-01

**Authors:** 


### ARTICLE X.

*Case of Malignant Disease of the Right Upper Jaw. Excision—Recovery.* Under the care of MR. F. LE GROS CLARK, St. Thomas' Hospital.

THE following case is one which affords strong encouragement to the operative surgeon. Not only did the patient recover well after a serious operation, by which she had been relieved of a very distressing and necessarily fatal disease; but, as several years have now elapsed since its occurrence, we are able to state that she remained well for a considerable period. Mr. Clark informs us that he saw the woman about six months ago, and that till shortly before that time she had been free from any indications of a return of the disease. There were, however, then symptoms of a very suspicious nature. She has not since presented herself. Although, therefore, it is to be feared that the cure will not prove permanent, yet somewhat more than two years of comfortable life have been obtained by the operation, a result most fully justifying its performance; and, considering the malignant nature of the disease, much better than could have been expected. With regard to excision of the upper maxilla, we may remark, that it is an operation very much more formidable in name than in reality. The case is in nowise exceptional to the general rule of such operations, in respect to the ease and rapidity with which the recovery was effected.

Mary Carter, æt. 53, married. Admitted into Lydia ward, St. Thomas' Hospital, November 29th, 1853.

From particulars furnished by Mr. Hare, of the Tunbridge Wells Infirmary, it appears that she was an inmate of that institution for some time during last summer. "She was admitted with a large fleshy tumor of the right upper jaw, which gave rise to inconvenience on account of its size; several teeth were removed, and the growth excised, after which potassa fusa was applied—the opinion of the surgeon being that the disease was malignant, and that eventually the jaw would have to be removed. She was so far relieved as to be able to leave the infirmary to all appearances well. She was discharged at her own request on September 17th, having been in since July 29th. On October 24th, she again applied for admission, and was made an out-patient; at this time the growth had resumed its original size; the whole of its exposed surface was ulcerated, and giving off a very fetid discharge: her general health was much impaired. Liq. potass. chlorid. wash, and an aliterative plan of treatment, was advised; she attended for three weeks only." This report brings the case down almost to the present date. From her statement to the reporter the following is gathered: that altogether she has been troubled with this affection for two years and a half—that at the commencement she perceived a very small nodulated swelling in the skin, as she says, over the canine fossa, which was loose and moved with the integument: this increased in size, slowly growing forwards, enlarging outwards, when she applied at the Tunbridge Wells Infirmary.

At the present time, November 29th, she has over her right cheek an elevated growth or tumor, with its surface uniform to the touch, and having a red, shiny appearance. Its situation is over the alveoli of the bicuspids and first molar. It is very soft and very tender when pressed. Examination of the interior of the mouth showed that the tumor extended from the gums to the cheek; the disease extending to the under part of the gums, but nowhere to the inner side of them. The free portion consisted of a non-ulcerated outer surrounding portion, and an interior ulcerated part, discharging a pus-like fluid, extremely fetid and offensive. Careful examination showed that the disease had extended to the superior maxilla; and accordingly the tumor was not movable.

She is not aware that any of her family ever suffered from anything like cancer. She has suffered for some time past from headache, and a severe aching pain along the angle of the lower jaw, and just under the ear of the affected side, which is increased on moving the jaws in mastication. With this exception her general health is pretty good; her appetite is fair, bowels regular, pulse good. Ordered—Ol. ricini, p. r. n.; lot. sod. chlorinat.

Mr. Clark, after two or three very careful examinations, decided on removing the tumor with a greater part of the maxilla. Mr. Green and Mr. Clark's colleagues saw the case, and agreed with him in this view. On December 10th, Mr. Clark performed the operation: he removed the tumor, part of the maxilla, leaving the floor of the orbit. The first incision was a long one in the axis of a line drawn from the root of the zygoma to the angle of the mouth; there was not much hemorrhage—no ligature required; the wound was brought together by means of harelip-pins. No chloroform used. She bore the operation well. In the evening of the same day the patient was quiet, and no hemorrhage from the wound. With hardly a bad symptom she convalesced rapidly; the night immediately after the operation she described as the most comfortable one she had had for months, which fact, however, is qualified by her taking at 10 P. M. 40 minims of the liq. opii sedativ. On the 29th she had a smart attack of diarrhea, which yielded readily to the ordinary astringents.

She left the hospital, January 10th, well.

Dr. Bristowe examined the tumor, and the following is an extract from his report:

Under the microscope it presented cells and nuclei of every size and shape; many of the cells were very large, and contained nuclei, also having within them nucleoli. The shape of the cells varied; their contents were faintly granular. The nuclei varied as much as the cells; sometimes they were free, and always contained one or more nucleoli. A few commencing characteristics of epithelial cancer were present, sufficiently distinct to show positively that the tumor belonged to that class.—*Ib.*

